# The role of the Quality and Outcomes Framework in the care of long-term conditions: a systematic review

**DOI:** 10.3399/bjgp17X693077

**Published:** 2017-09-26

**Authors:** Lindsay JL Forbes, Catherine Marchand, Tim Doran, Stephen Peckham

**Affiliations:** Centre for Health Services Studies, University of Kent, Canterbury, Kent.; Centre for Health Services Studies, University of Kent, Canterbury, Kent.; Department of Health Sciences, University of York, York.; Centre for Health Services Studies, University of Kent, Canterbury, Kent.

**Keywords:** incentive reimbursement, primary health care, quality indicators, health care, quality of health care

## Abstract

**Background:**

Improving care for people with long-term conditions is central to NHS policy. It has been suggested that the Quality and Outcomes Framework (QOF), a primary care pay-for-performance scheme that rewards practices for delivering effective interventions in long-term conditions, does not encourage high-quality care for this group of patients.

**Aim:**

To examine the evidence that the QOF has improved quality of care for patients with long-term conditions.

**Design and setting:**

This was a systematic review of research on the effectiveness of the QOF in the UK.

**Method:**

The authors searched electronic databases for peer-reviewed empirical quantitative research studying the effect of the QOF on a broad range of processes and outcomes of care, including coordination and integration of care, holistic and personalised care, self-care, patient experience, physiological and biochemical outcomes, health service utilisation, and mortality. Because the studies were heterogeneous, a narrative synthesis was carried out.

**Results:**

The authors identified three systematic reviews and five primary research studies that met the inclusion criteria. The QOF was associated with a modest slowing of both the increase in emergency admissions and the increase in consultations in severe mental illness (SMI), and modest improvements in diabetes care. The nature of the evidence means that the authors cannot be sure that any of these associations is causal. No clear effect on mortality was found. The authors found no evidence that the QOF influences integration or coordination of care, holistic care, self-care, or patient experience.

**Conclusion:**

The NHS should consider more broadly what constitutes high-quality primary care for people with long-term conditions, and consider other ways of motivating primary care to deliver it.

## INTRODUCTION

The UK’s Quality and Outcomes Framework (QOF) is the world’s largest pay-for-performance scheme in primary care. It rewards general practices financially for delivering interventions and achieving patient outcomes using evidence-based indicators developed by the National Institute for Health and Care Excellence (NICE).^1^ Although the QOF is voluntary, nearly 99% of practices in England participate, on average deriving 10–15% of total practice income from the scheme.^2^

The introduction of the QOF in 2004 was a part of a new national contract for GPs, driven by the need to respond to years of underinvestment in general practice compared with other parts of the health service, low morale among GPs, and variations in the quality of primary medical care.^3^^,^^4^ The QOF was intended to provide a mechanism to motivate GPs and to increase funding for their practices, and the vast majority of practices took up the opportunity for additional income. Evidence from the early years of the scheme suggested it reduced variations between practices in the delivery of incentivised interventions,^5^ and contributed to progress towards better use of electronic records and nurse-led multidisciplinary care of long-term conditions.^3^ After the first year of the QOF, most practices achieved near-maximum remuneration from the scheme.^2^

Arguably, then, the QOF achieved what it set out to do. But this may have come at a cost. It has been suggested that practices prioritise QOF-related activities at the expense of other aspects of care, because of their reliance on QOF income.^6^^,^^7^

A decade after the introduction of the QOF, NHS strategy, set out in the 2014 *Five Year Forward View*,^8^ is now focused on other challenges. These include finding new ways to manage people with long-term conditions, whose care is estimated to consume 70% of health service resources.^8^ Most clinical QOF indicators relate to the care of long-term conditions and are based on good evidence,^4^ but tend to be limited in scope, focusing on single, biomedical dimensions of care. Appendix 1 provides a brief description of the 68 QOF indicators relating to care of long-term conditions in 2016–2017; the total number of indicators for that year was 77.

In 2015, the Royal College of General Practitioners called for the replacement of the QOF to allow GPs *‘to focus on providing the best possible holistic care’*.^9^ NHS England, in April 2016, undertook to review the QOF, acknowledging that it may have *‘served its purpose’* and may be *‘a barrier to holistic management’*.^10^ In early 2017, the British Medical Association called for the QOF to be suspended to reduce bureaucratic pressures and free up clinical time.^11^ Scotland abolished the QOF in 2016.^3^

The Policy Research Unit in Commissioning and the Health Care System was commissioned to undertake a review, led by the Centre for Health Services Studies at the University of Kent, to report in September 2016. The authors aimed to examine the evidence that the QOF has improved care and outcomes for patients with long-term conditions, including elements of care highlighted as priorities in the *Five Year Forward View*,^8^ such as coordinated and integrated care, holistic and personalised care, and self-care.

**How this fits in**

The usefulness of the Quality and Outcomes Framework (QOF) as a tool for promoting progress towards the vision of the *Five Year Forward View* for care of long-term conditions has been questioned. This systematic review found no convincing evidence that the QOF can promote better integrated care, personalised, holistic care, or self-care — or, indeed, improve any other outcomes in people with long-term conditions. The NHS should consider other ways of supporting general practice to deliver the vision of the *Five Year Forward View*.

## METHOD

The authors searched for reports of empirical quantitative research examining the effectiveness of the QOF in the management of long-term conditions, published in peer-reviewed journals in English. They included studies of populations registered with GPs in the UK, and excluded studies of locally designed and implemented pay-for-performance schemes, and studies of limited geographical scope (which were defined as examining data from fewer than four primary care trusts in England, or fewer than 100 practices in Scotland) because of likely low generalisability. The authors included studies where the comparator was any other method of funding general practice, concurrent or historical, and, if there was no concurrent comparator, where the analysis controlled for underlying trends. They set no limits on outcomes except that they were measured quantitatively and related to patients with long-term conditions, including:

- measures of health or morbidity: biochemical and physiological measures, mortality, hospital admissions;
- biomedical aspects of delivery of care: diagnostics, plans, referrals, and ongoing monitoring, clinical interventions (for example, prescriptions, immunisations), consultation rates;
- broader aspects of care: coordination, continuity or integration of care, holistic care (that is, that considers multiple morbidity and social context, personalised for the patient), self-care; and
- patient perspectives: patient experience, quality of life, or satisfaction.

The authors included randomised controlled trials, longitudinal studies where the analysis attempted to control for underlying trends, controlled before- and-after studies, and systematic reviews of these. They excluded cross-sectional studies examining how outcomes varied according to QOF achievement, because of the lack of suitable controls, the near-universal high level of achievement, and the high likelihood of confounding of associations between QOF achievement and outcome by other factors. The authors also excluded studies in which the researchers estimated or modelled outcomes rather than reporting empirical data (more details of the inclusion and exclusion criteria are available from the authors). The authors searched electronic databases (Cochrane Database, Medline, Embase, and Health Management Information Consortium) for studies published between 2004 (the year the QOF was introduced) and May 2016 (see Box 1 for search terms). They examined references of identified papers to search for further reports and asked experts for references to other relevant research.

Box 1. **Search terms**

**Search 1**
- Quality Outcomes Framework (keyword) OR
- Quality and Outcomes Framework (keyword OR
- QOF (keyword)

**Search 2**
- Pay-for-performance (keyword) or Reimbursement (Medical Subject Heading (MeSH) term) AND
- Primary health care (keyword) or Primary Health Care (MeSH term) OR
- Primary medical care (keyword) or Family practice (MeSH term) OR
- General practice (keyword) or General Practice (MESH term)

Two of the authors assessed suitability for inclusion for each abstract identified, and, where there was no consensus, asked a third author to adjudicate. Data were extracted independently from all papers by two authors.

The authors assessed quality of randomised controlled trials using adaptations of the Cochrane Collaboration’s tool,^12^ and longitudinal studies and systematic reviews using tools adapted from those developed by the National Institutes of Health.^13^^,^^14^

## RESULTS

### Identification and description of studies

**Figure 1.**
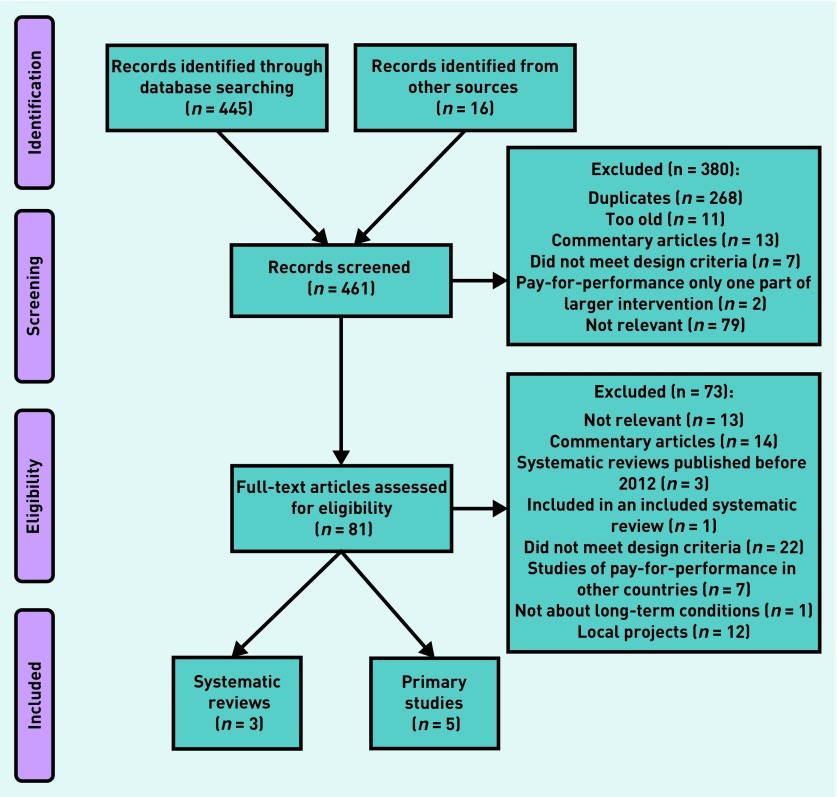
***Search process and results.***

Figure 1 shows the process of identification of studies. The three most recent systematic reviews asking the same questions as this review had search dates in 2012^15^^,^^16^ and 2015.^17^ These included 20 studies of the QOF in total.^18^^–^37 The systematic review with the 2015 search date^17^ identified two studies of the QOF,^22^^,^^32^ both of which had been published in 2011 and had been included in one or other of the two reviews with search dates in 2012.

In all three reviews, while the authors set few limits on outcomes, study outcomes were solely derived from either QOF indicators themselves or prescribing data, with two exceptions: one study examined adherence to British Thoracic Society spirometry standards^36^ and the other cardiovascular events in patients with hypertension.^32^

The authors found five primary research studies meeting the search criteria published since 2012.^38^^–^42 The first examined trends in mortality rates for conditions covered by the QOF, comparing the UK with other high-income countries with no pay-for-performance schemes in primary care.^38^ The others examined the effect of introducing the QOF on patient management and outcomes, either nationally,^39^ or in samples of practices participating in the UK General Practice Research Database (GPRD) or Clinical Practice Research Datalink (CPRD) (627 UK practices,^40^ 516 UK practices,^41^ 148 English practices^42^).

The studies’ outcomes were: mortality from long-term conditions;^38^ hospital admissions for a range of QOF and non-QOF conditions;^39^ consultation rates in severe mental illness (SMI);^40^ prescribing in type 2 diabetes;^41^ and a composite indicator derived from QOF data on processes and outcomes of care in type 2 diabetes.^42^ Because of a high degree of study heterogeneity, the authors carried out a narrative synthesis. Table 1 summarises the design and results of primary research studies.

**Table 1.** Primary research studies examining the effect of QOF on patient management and outcomes

**Authors**	**Year of publication**	**Geographical area and period under study**	**Main outcome**	**Key results**
Ryan A, *et al* 38	2016	Populations of UK and 27 other high income countries,1994–2010.	Mortality levels from chronic diseases targeted by QOF.	Mortality fell in all countries over the period. QOF not associated with any step change in mortality in the UK. Difference between mortality/100 000 between UK observed and expected −3.7 (95% CI = −8.2 to 0.8).
Harrison MJ, *et al* 39	2014	All English practices, 1998–2010.	Rates of emergency admissions: that can be prevented in the community, including those for — conditions for which care incentivised in QOF — conditions for which care not incentivised in QOF that cannot be prevented in community care.	Emergency admission rates increased by 34%, but rate of increase lower for conditions for which care incentivised by QOF than other types of emergency admission. In 2003, no difference in emergency admission rates between those for conditions for which care is incentivised by QOF and those for which care not incentivised by QOF. By 2010, rates of emergency admissions for conditions for which care is incentivised by QOF 8% (95% CI = 6.9 to 9.1) lower than those for which care is not incentivised by QOF. By 2010, rates of emergency admissions for conditions for which care is incentivised by QOF 11% (95% CI = 10.1 to 11.7) lower than those that cannot be prevented. The lower increase in emergency admission rates among those for whom care incentivised by QOF was mainly driven by admissions for coronary heart disease.
Kontopantelis E, *et al* 40	2015	A total of 627 practices in the CPRD across UK, 2000–2011.	Primary care consultation rates in people with SMI and people without SMI.	Mean consultation rates between 2000 and 2011: in SMI — increased from 22 to 49 per year (92% increase); people without SMI — increased from 10 to 19 per year (75% increase). For both, trend of increasing rates before 2004. Significant step change increase in 2004 for both groups, much bigger step change for people with SMI. After this, rate of increase declined in both groups. Face-to-face consultation rate: in people with SMI, nine/patient/year 2000–2003, rising to 11/patient/year in 2011;. in people without SMI, about five/patient/year over the whole period.
Gallagher N, *et al* 41	2015	A total of 516 practices in the GPRD across UK, 1999–2008.	The % of newly diagnosed patients with type 2 diabetes prescribed medication within 24 months after diagnosis.	Pre-intervention 1999–2003: rate decreasing by 1.4% per year (95% CI = 0.8 to 2.1). Post-intervention 2004–2008: rate increased by 1.6% per year (95% CI = 0.8 to 2.3).
Kontopantelis E, *et al* 42	2013	A total of 148 practices in the GPRD across England, 2001–2006.	Delivery of care of type 2 diabetes — composite of achievement of the 17 diabetes QOF indicators, including processes and outcomes.	Pre-intervention 2001–2003: delivery of care improving. Post-intervention 2004–2006: delivery of care improved over and above the previous trend. In the first year, 14% improvement in score over and above expected (95% CI = 13.7 to 14.6). By third year, 7% improvement in score over and above expected (95% CI = 6.7 to 8.0).

CI = confidence interval. CPRD = Clinical Practice Research Datalink. GPRD = General Practice Research Database. QOF = Quality and Outcomes Framework. SMI = severe mental illness.

None of the relevant studies identified by the systematic reviews, or the primary studies published since 2012, examined the effect of the QOF on broader aspects of care or patient perspectives.

### Quality

The systematic reviews were of good quality. Due to the nature of the intervention, the primary research studies were all before- and-after studies using interrupted time series or difference-in-differences methods and, as such, were of good quality for observational studies. However, because of the study designs, the authors cannot be sure that the QOF was responsible for any change in outcomes.

### Findings

#### Systematic reviews

The first systematic review concluded that the QOF had had a limited impact on health outcomes.^15^ The second systematic review concluded that the effect of pay-for-performance remained uncertain.^16^ The third systematic review concluded that there was limited evidence of the effects of financial incentives.^17^

#### Primary research

The study examining trends in mortality in the UK compared with other countries found no effect of the QOF, although the synthetic control approach^43^ adopted in the study required the use of conservative tests for statistical inference.^38^ The study examining emergency admissions before and after the introduction of the QOF found that the trend of increasing emergency hospital admission rates (which increased overall by 34% between 2004 and 2010) was modestly lower for conditions incentivised in the QOF compared with conditions that were not incentivised in the QOF, by 3% in the first year rising to 8% in 2010.^39^ The difference was mainly driven by relative reductions in emergency admission rates for coronary heart disease.

The study examining consultation rates found a trend of increasing rates overall during the period, with a small step change in 2004; the rate of increase was greater in people with SMI than overall.^40^ The face-to-face consultation rate in SMI increased from about nine to 11 per patient per year from 2000 to 2011, and in other people it stayed stable at about five per patient per year over the same period.

The study of prescribing in type 2 diabetes found a modest increase in prescribing of antidiabetic medication (changing the direction of the trend of decreasing initiation rates to increasing initiation rates) after the introduction of the QOF.^41^ The increase was sustained at a similar rate until 2008.

The study examining effects on a composite indicator of process and outcomes in type 2 diabetes found a modest improvement of 14% over and above the underlying trend in the first year after the introduction of the QOF, declining to 8% in the third year.^42^ Table 1 summarises the results of the studies.

## DISCUSSION

### Summary

The authors found evidence that the QOF may be associated with a modest reduction in emergency admission rates in long-term conditions, a modest increase in consultation rates in SMI, and modest improvements in certain limited aspects of the care of diabetes. They found no clear evidence that these changes have led to any effect on mortality. Because of the design of the studies, it is not possible to be sure that any of the positive effects seen are causally related to the QOF.

The authors found no evidence to suggest that the QOF influences, positively or negatively, other aspects of care, such as integration or coordination of care, holistic or personalised care, or self-care, nor any evidence of its effects on patients’ quality of life, experience, or satisfaction.

The QOF is unlikely to advance progress towards the vision of the *Five Year Forward View* for the care of long-term conditions. To deliver the aims of the *Five Year Forward View*, the NHS should consider more broadly — beyond what is measured by the QOF — what constitutes high-quality primary care for people with long-term conditions, and consider managing performance on this basis. In the context of a demoralised primary care workforce, it is important also to consider ways other than financial incentives to motivate primary care teams to deliver high-quality care.

### Strengths and limitations

To the authors’ knowledge, this review is the first to have specifically addressed the effect of the QOF on those aspects of care for long-term conditions that are prioritised by national policy. As with any systematic review, the authors’ conclusions are constrained by the limited quantity and quality of the primary research published to date. Although the search for quantitative research was comprehensive, the authors did not include qualitative research, which may provide other insights.

Research to date has not attempted to identify the effects of the QOF on any of the broader aspects of care for long-term conditions, having examined effects only on more easily measurable outcomes, for example, those collected as part of the QOF, or routinely available data on mortality, emergency admissions, consultation rates, and prescribing. The authors found no evidence of attempts to evaluate the QOF using validated measures of quality of care in general practice. Perhaps this is because defining and measuring quality of general practice is complex.^44^

The lack of effect of the QOF on mortality is surprising, given that the indicators are based on high-quality evidence of effectiveness of interventions. Why this is the case is not clear. The wider determinants of population health (including low income, experience of inequality or discrimination, or quality of air, education, housing, or work conditions^45^) may be much more important than the quality of care in determining mortality. Also, it is recognised that the effectiveness of interventions demonstrated in randomised controlled trials, which include highly selected study participants, is often diluted in routine clinical practice.^46^ Perhaps non-incentivised activities are more important in determining mortality in the patient population. It is also possible that practices misreport performance so as to exaggerate the quality of care, although there is little evidence that this is a significant problem.^47^

The authors found evidence that the QOF was associated with a modest slowing of the increase in emergency admissions for conditions for which care is incentivised by the QOF, and an increase in primary care attendances for people with serious mental illness. Whether the QOF is responsible for these is unclear; many other factors are likely to have influenced admission and attendance rates over the period, including changes in medical technology or access to other services, or national standards for management of long-term conditions. In any case, among interventions to prevent emergency admissions, pay-for-performance is unlikely to be one of the most effective.^48^^,^^49^

It could be argued that some QOF indicators — in palliative care, cancer, SMI, dementia, and rheumatoid arthritis — incentivise multidisciplinary meetings, reviews, and care plans, considered necessary elements of holistic and integrated care (as set out in recent guidance from NICE).^50^ However, to achieve the indicators, practices are not required to demonstrate that their activities ensure that holistic or integrated care has been delivered.

### Implications for research and practice

The authors found no convincing evidence that the QOF promotes better care and outcomes for people with long-term conditions. QOF may also have negative effects. If practices have achieved maximum or near maximum points under the scheme (which is true for most practices), they have little motivation to improve achievement further. It is likely that the QOF diverts practices and professionals from ways of providing high-quality primary care that is not QOF-related. Moreover, the QOF does not incentivise practices to improve care for patients with the most complex needs in primary care, because these are more likely to be excepted from the scheme.^51^ Raising thresholds for achievement may be counterproductive — there is evidence that it leads to increased exception reporting, raising apparent achievement with no real increase in the desired activity.^52^

The Chief Executive of the NHS announced in October 2016 that the QOF would be phased out.^53^ What would happen to the quality of primary care if the QOF is completely abolished is not clear, although it seems unlikely that standards would drop significantly, because the activities rewarded in the QOF are now firmly embedded in practice. There is some limited evidence to suggest that performance did not fall following the withdrawal of certain individual indicators from the scheme.^54^ Abolishing the QOF may also allow practices to prioritise other activities, which could lead to better care.

The QOF provides a major component of practice income; if it were abolished, practices would need to be assured of a stable income. Losing this is likely to have detrimental effects on patient care and further worsen recruitment and retention in primary care, which is once again in a precarious position.^55^

Alternative methods of rewarding good practice are being considered for new models of primary care.^56^ Any replacement for the QOF needs to consider the evidence of effectiveness of pay-for-performance in primary care, and the evidence of what motivates primary care professionals to provide high-quality care.^57^

## Appendix 1. QOF indicators 2016/2017 relating to care of long-term conditions

**Table:**

**Long-term condition**	**QOF code**	**Brief description of indicator**
Asthma	AST001	Register of patients with asthma
	AST002	Percentage of patients with asthma and measures of variability or reversibility recorded
	AST003	Percentage of patients with asthma who have had control assessed
	AST004	Percentage of patients with asthma with record of smoking status

Atrial fibrillation	AF001	Register of patients with atrial fibrillation
	AF006	Percentage of patients with atrial fibrillation in whom stroke risk has been assessed
	AF007	Anticoagulant therapy in those with atrial fibrillation and high risk of stroke

Cancer	CAN001	Register of patients with cancer
	CAN003	Percentage of patients with cancer who have been reviewed

Chronic kidney disease (CKD)	CKD001	Register of patients with chronic kidney disease

Chronic obstructive pulmonary disease (COPD)	COPD001	Register of patients with COPD
	COPD002	Percentage of patients with COPD with diagnosis confirmed by post-bronchodilator spirometry
	COPD003	Percentage of patients with COPD who have had a review with assessment of breathlessness
	COPD004	Percentage of patients with COPD with a record of forced expiratory volume in 1 second (FEV1)
	COPD005	Percentage of patients with severe COPD with record of oxygen saturation
	COPD007	Percentage of patients with COPD who have had influenza immunisation

Coronary heart disease (CHD)	CHD001	Register of patients with CHD
	CHD002	Percentage of patients with CHD with blood pressure 150/90 mmHg or less
	CHD005	Percentage of patients with CHD taking aspirin, an alternative antiplatelet therapy, or an anticoagulant
	CHD007	Percentage of patients with CHD who have had influenza immunisation

Dementia	DEM001	Register of patients with dementia
	DEM004	Percentage of patients with dementia whose care plan has been reviewed face-to-face
	DEM005	Percentage of patients with a new diagnosis of dementia with record of tests to exclude reversible cause

Depression	DEP003	Percentage of patients with new diagnosis of depression with review soon after diagnosis

Diabetes mellitus	DM002	Percentage of patients with diabetes with blood pressure 150/90 mmHg or less
	DM003	Percentage of patients with diabetes with blood pressure 140/80 mmHg or less
	DM004	Percentage of patients with diabetes with total cholesterol 5 mmol/l or less
	DM006	Percentage of patients with diabetes and nephropathy taking angiotensin converting enzyme inhibitors or angiotensin receptor blockers (ACEIs or ARBs)
	DM007	Percentage of patients with diabetes with glycosylated haemoglobin 59 mmol/mol or less
	DM008	Percentage of patients with diabetes with glycosylated haemoglobin 64 mmol/mol or less
	DM009	Percentage of patients with diabetes with glycosylated haemoglobin 75 mmol/mol or less
	DM012	Percentage of QOF patients with diabetes with a record of a foot examination and foot risk classification
	DM014	Patients newly diagnosed with diabetes referred to a structured education programme
	DM017	Register of patients with diabetes
	DM018	Percentage of patients with diabetes who have had influenza immunisation

Epilepsy	EP001	Register of patients with epilepsy

Heart failure	HF001	Register of patients with heart failure
	HF002	Percentage of patients with heart failure confirmed by an echocardiogram or by specialist assessment
	HF003	Percentage of patients with heart failure taking ACEIs or ARBs
	HF004	Percentage of patients with heart failure taking ACEIs or ARBs plus beta-blocker

Hypertension	HYP001	Register of patients with hypertension
	HYP006	Percentage of patients with hypertension with blood pressure of 150/90 mmHg or less
	CVD-PP001	Percentage of patients with hypertension and high cardiovascular risk treated with statins

Learning disability	LD003	Register of patients with learning disability

Mental health	MH001	Register of patients with serious mental health problems
	MH002	Percentage of patients with serious mental health problems with comprehensive care plan
	MH003	Percentage of patients with serious mental health problems with record of blood pressure
	MH007	Percentage of patients with serious mental health problems with record of alcohol consumption
	MH008	Percentage of women with serious mental health problems with cervical screening test performed
	MH009	Percentage of patients on lithium therapy having renal and thyroid function monitored
	MH010	Percentage of patients on lithium therapy with lithium levels in therapeutic range

Osteoporosis	OST002	Percentage of patients 50–74 with confirmed osteoporosis taking bone-sparing agent
	OST004	Register of patients with osteoporosis
	OST005	Percentage of patients aged >75 with osteoporosis taking bone-sparing agent

People with palliative care needs	PC001	Register of patients in need of palliative care/support
	PC002	Regular multidisciplinary case review meetings for people receiving palliative care

Peripheral arterial disease	PAD001	Register of patients with peripheral arterial disease
	PAD002	Percentage of patients with peripheral arterial disease with blood pressure 150/90 mmHg or less
	PAD004	Percentage of patients with peripheral arterial disease taking aspirin or an alternative antiplatelet

Rheumatoid arthritis	RA001	Register of patients with rheumatoid arthritis
	RA002	Percentage of patients with rheumatoid arthritis who have had a face-to-face review

Stroke or transient ischaemic attack (STIA)	STIA001	Register of patients with STIA
	STIA003	Percentage of patients with STIA with blood pressure 150/90 mmHg or less
	STIA007	Percentage of patients with non-haemorrhagic stroke or TIA taking antiplatelet agent, or anticoagulant
	STIA008	Percentage of patients with STIA referred for further investigation
	STIA009	Percentage of patients with STIA who have had influenza immunisation
**Several long-term conditions**	SMOK002	Percentage of patients with long-term conditions with record of smoking status
	SMOK005	Percentage of smokers with long-term conditions offered smoking cessation support

QOF = Quality and Outcomes Framework.
